# Sustainable Mixed-Halide Perovskite Resistive Switching Memories Using Self-Assembled Monolayers as the Bottom Contact

**DOI:** 10.1021/acs.jpclett.4c01664

**Published:** 2024-07-22

**Authors:** Michalis Loizos, Konstantinos Rogdakis, Emmanuel Kymakis

**Affiliations:** † Department of Electrical & Computer Engineering, Hellenic Mediterranean University (HMU), Heraklion 71410, Crete, Greece; ‡ Institute of Emerging Technologies, University Research and Innovation Center, HMU, Heraklion 71410, Crete, Greece

## Abstract

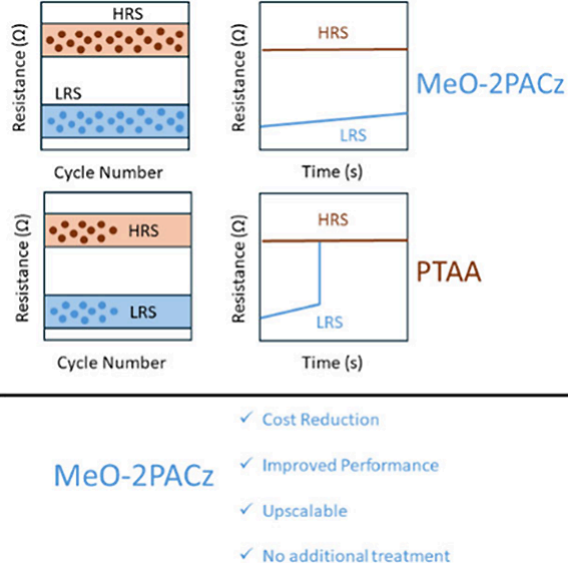

The complex ionic-electronic conduction in mixed halide perovskites enables their use beyond von Neumann architectures implemented in resistive switching memory devices. Although device fabrication based on perovskite compounds involves solution-processing at low temperatures, reducing further fabrication costs by eliminating expensive materials can improve their compatibility with upscalable deposition techniques. Notably, the substrate on which the perovskite active layer is developed has been reported to severely affect its quality and thus the overall device performance. Hereby, we demonstrate the sustainable manufacturing of memristive perovskite solar cells by replacing the expensive poly[bis(4-phenyl)(2,4,6-trimethylphenyl)amine] (PTAA) that serves as a hole transporting layer (HTL) with a self-assembled monolayer (SAM), namely [2-(3,6-dimethoxy-9*H*-carbazol-9-yl)ethyl]phosphonic acid (MeO-2PACz). Multiple sequential memristive current–voltage characteristics of single devices are reported, and average data of multiple reference and targeted devices are compared. Resistive switching memory devices based on SAM exhibit improved performance having reduced average SET voltage values and narrower statistical variation compared to reference devices with PTAA. It is shown that both PTAA and SAM based devices exhibit high ON/OFF ratio of about 10^3^ operating at low switching electric fields. Replacing an expensive polymer-based HTL with this approach reduces fabrication costs compared to PTAA.

The emergence of optoelectronic devices based on mixed halide perovskites (MHP) is the result of their peculiar and advantageous semiconductive properties. Extensive research on perovskite solar cells (PSCs) has led to efficiencies above 26%.^1−9^ The usage of halide perovskites has been recently demonstrated in a variety of optoelectronic device applications going far beyond solar energy harvesting.^10,11^ One notable example is MHP’s application in resistive switching (RS) memories that are emerging devices employed for in-memory neuromorphic computing. Neuromorphic and RS devices based on MHP is an appealing direction due to their mixed ionic-electronic conduction^12^ that enables ion migration and hysteresis associated with the onset of the RS effect.^13^

Mixed halide perovskites (MHP) consist of the formula ABX_**3**_, with X being an anion such as iodine (I^–^), bromide, or chloride (Cl^–^), B site is a divalent cation occupied by typically Pb^2+^, and the A site cation is monovalent and is either organic like formamidinium (FA^+^) or methylammonium (MA^+^) or inorganic like Rb or Cs. Modifications in this structure affect several properties of the perovskite films. For instance, using multiple A site cations resulted in high efficiency PSCs with improved stability.^14−16^ Furthermore, mixing iodine, bromide, and chloride can tune the bandgap of the perovskite film.^17^ These properties can be beneficial for the case of RS memories.^18^ MHP RS memories with multiple cations can increase the cycling endurance,^19^ while the incorporation of rubidium enhances RS performance as it can narrow the growth of the conductive filament.^20^ In our previous work, we have shown that an MHP device with a photovoltaic structure could have concurrently RS characteristics, and thus the resulting device could possess both memristive and energy harvesting properties.^21^ For these dual mode RS devices, an inverted solar cell structure was shown to be critical, rendering the role of the hole transporting layer (HTL), which affects the quality of the perovskite layer, very important.

MHP were successfully exploited as nonvolatile resistive switching memories exhibiting good figure of merit such as a high ON/OFF ratio,^22,23^ fast switching speed,^24^ and good state retention and cycling endurance.^25^ Going from single memristive devices to multiple devices integrated in a crossbar array, optoelectronic^26,27^ MHP-based artificial synapses also have been demonstrated opening the path for brain-inspired in-memory computing,^28,29^ at low power consumption^30,31^ contributing to designing neuromorphic computing architectures^32^ for IoT edge sensing devices.^33,34^ Apart from synapses implementation, the transient ion dynamics of perovskites enabled by diffusive/volatile memristive devices^35^ is shown as a convenient tool for emulating neuron dynamics^36^ with the facilitation of theoretical modeling.^37−41^ Despite the miscellaneous assets of perovskites that involve low-temperature solution processing and compatibility with flexible substrates, achieving durable and reliable nonvolatile memory behavior remains a challenge as MHP memories suffer from relatively poor cycling endurance.^13^ An approach to address this issue is to assemble 2D/3D heterostructures, which enhances the RS performance.^42,43^

Bottom contacts are important to facilitate sufficient hole transfer and injection in PV applications as well as producing uniform, high quality films, which is essential for reliable and high-performing RS memory devices. Poly[bis(4-phenyl)(2,4,6-trimethylphenyl)amine] (poly(triarylamine), PTAA) is a common material^44^ used as a hole transporting layer (HTL)^14,45^ in inverted PSCs resulting in high-performing PSCs in its pristine form,^46−48^ or upon modifications.^49,50^ However, PTAA is a relatively expensive material, and it is hydrophobic, which presents challenges to achieving uniform perovskite films deposited on it, targeting either PV or RS applications. These PTAA drawbacks that complicate device fabrication promote alternative materials development targeting lower manufacturing costs and more sustainable processes. An example of a bottom layer for a uniform RS MHP layer is the case of PEDOT:PSS, which assists in forming uniform perovskite films for stable RS. Ismail et al. introduced an ultrathin amorphous zinc tin oxide (ZTO) layer between the ZrO_2_ active layer and the TiN bottom electrode, resulting in more uniform and reproducible RS with multilevel states.^51^ George and Arumugam Vadivel Murugan incorporated an HfO_2_-doped TiO_2_ and Al_2_O_3_ layer between the fluorine-doped tin oxide (FTO) bottom electrode and the CH_3_NH_3_PbI_3_ perovskite, an approach that reduced the operating voltage of the RS device.^52^ Nevertheless, more research is necessary to emphasize the role of the bottom buffer layer in the RS characteristics.

These issues can be resolved by introducing self-assembled monolayer molecules (SAMs). SAMs are ultrathin films composed of ordered arrays of organic molecules that are spontaneously assembled on the surface of the substrate. The SAM molecules have functional groups that allow them to bind to the substrate. The thermodynamically stable formation of the monolayer depends on the chemical adsorption of the molecules on the surface of the substrate and on the intermolecular interactions.^53^ SAMs consist of three main parts: The headgroup/anchoring group interacts chemically with the surface allowing the molecules to attach on the surface, while the spacer/linkage group controls the assembly process of the molecules through van der Waals interactions that determine the optoelectronic properties of the SAM.^54^ Finally, the functional/terminal group determines the properties and surface morphology of the resulting ultrathin film, enabling tunable functionality depending on the chemical group used, which can lead to several device applications. As a consequence, SAM molecules can improve the interface properties with the resulting perovskite film, thus passivating interfacial defects.^55,56^ These properties extend the application of SAMs in electronic devices such as LEDs,^57^ transistors,^58^ solar cells,^59^ and sensors.^60^ In addition, SAM molecules are able to reduce traps and voids in the perovskite layer resulting to enhanced interface properties when compared to the perovskite films grown on PTAA.^61−64^ SAM molecules are also successfully employed in large-area devices.^56,65−68^ Common SAM molecules used in PSCs^69^ include [2-(3,6-dimethoxy-9*H*-carbazol-9-yl)ethyl]phosphonic acid (MeO-2PACz) and [2-(9*H*-carbazol-9-yl)ethyl]phosphonic acid, although other derivatives have been developed as well.^62,70,71^

Herein, we report a cost-effective method to improve the performance of MHP based RS memories based on an inverted memristive solar cell structure with an ON/OFF ratio of >10^3^ and low voltage operation window by introducing the self-assembled monolayer MeO-2PACz as a replacement for the expensive PTAA HTL. Experimental evidence based on electrical pulses measurements show that the MeO-2PACz memristive device exhibits improved cycling endurance of 10^4^ cycles and extended 3 × 10^3^s retention compared to the PTAA device. The improved interface of the SAM with the perovskite layer is likely responsible for the improved performance of target devices and the observed reduction of the SET voltage. The proposed methodology enables the sustainable fabrication of both scalable and printable MHP memristive devices with a lower manufacturing cost.

To elucidate the effect of the SAM on the switching characteristics of the perovskite memories, we fabricated devices based on a glass/ITO/HTL/Cs_0.05_Rb_0.04_(FA_0.85_MA_0.15_)_0.91_Pb(I_0.85_Br_0.15_)_3_ (MA = methylammonium, FA = formamidinium)/phenyl-C61-butyric acid methyl ester (PC_60_BM)/2,9-dimethyl-4,7-diphenyl-1,10-phenanthroline (bathocuproine, BCP)/Ag configuration, where HTL is the bottom contact and was either poly[bis(4-phenyl)(2,4,6-trimethylphenyl)amine] (poly(triaryl amine), PTAA) or SAM, namely [2-(3,6-dimethoxy-9*H*-carbazol-9-yl)ethyl]phosphonic acid (MeO-2PACz), as depicted in Figure 1b. All experiments for the SAM and PTAA devices were performed in a N_2_-filled glovebox. Both HTL materials were deposited by spin-coating on ITO-coated glass substrates and were subsequently annealed at 100 °C for 10 min. The thickness of the resulting PTAA and SAM film was estimated at 12.7 ± 2.0 and 3.7 ± 1.2 nm, respectively, by AFM measurement (Figure S1). The perovskite was deposited using a high-speed single dynamic spin-coating step at 6000 rpm, following a chlorobenzene antisolvent treatment. The samples were then annealed at 100 °C for 45 min. Figure S2 shows a 5 μm × 5 μm image of the polycrystalline perovskite film. The resulting perovskite film is uniform, without pinholes, which is important for stable RS characteristics. The thickness of the obtained perovskite film is ∼380 nm.^72^ Afterward, the PCBM and BCP solutions are spin-coated on top of the HTL/perovskite films, following the thermal evaporation of the Ag top electrode. The fabrication process is illustrated in Figure 1a. More details about device fabrication and characterization can be found in the Supporting Information. It is worth mentioning that the proposed inverted photovoltaic device configuration has demonstrated dual energy harvesting and photonic in-memory computing abilities with synaptic functionalities according to our previous works.^21,73^ The optoelectronic switching properties of our device enabled the design of a neural network with a reduced number of synapses toward energy-efficient and reduced perplexity neuromorphic computing.^74^

**Figure 1:**
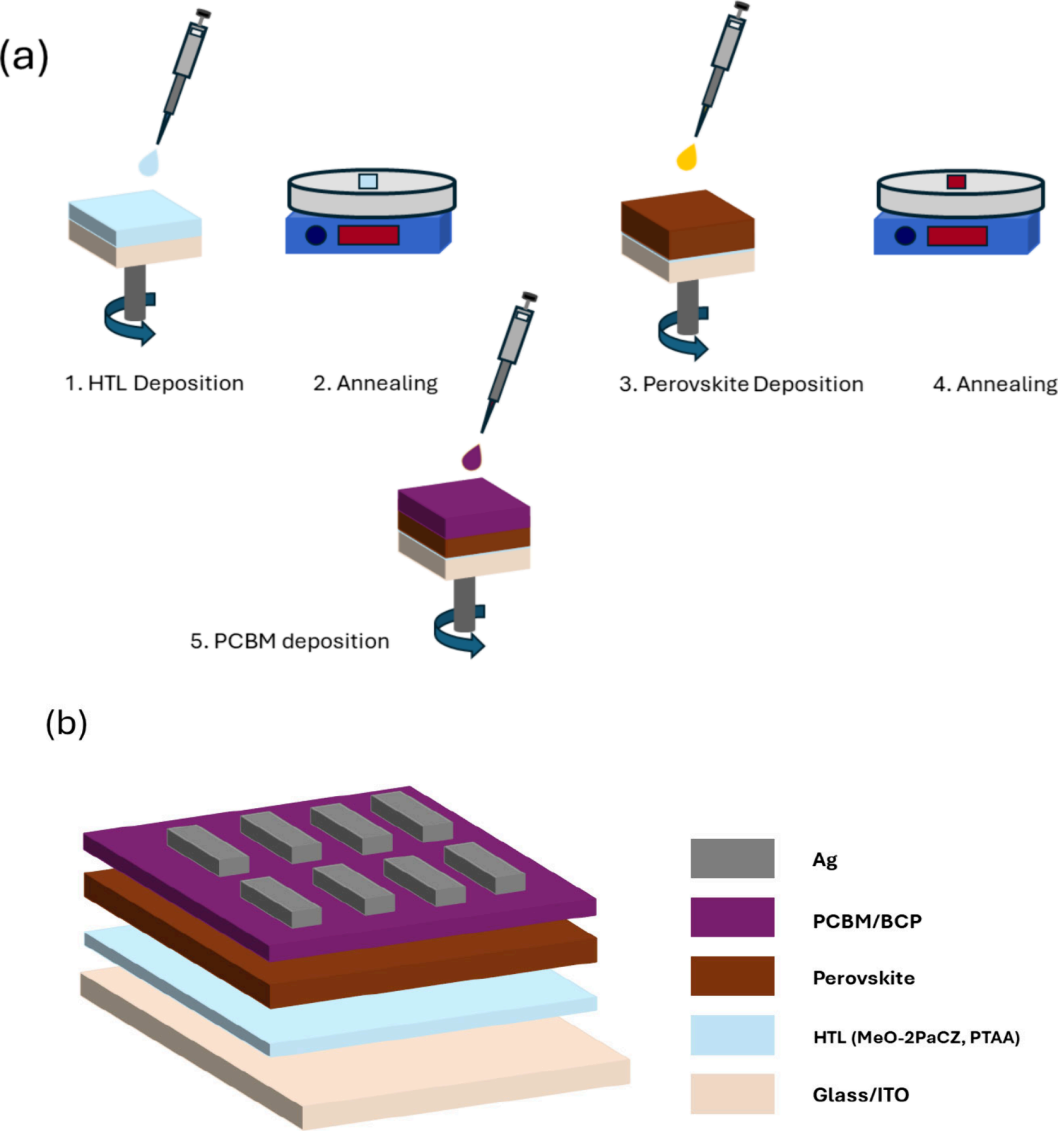
a) Process flow for the fabrication of perovskite resistive switching memory. b) Schematic illustration of the resulting device.

The steady-state *I*–*V* characteristics of the MHP resistive switching memory devices for both HTLs were investigated by sweeping the voltage from a positive to a negative bias. The device properties with both HTLs are shown in Figure 2a,b for 20 consecutive current–voltage sweeps for a single cell. The SET process occurs at a positive bias, while the RESET process occurs at a negative bias, confirming that both types of MHP devices possess bipolar resistive switching characteristics, with an operation window at ±1 V for both the SET and RESET process. It should be mentioned that during all measurements, a compliance current of 10 mA, scan rate of 100 mV s^–1^, and 10 mV step was used. According to our previous work, varying the scan rate from 10 to 100 mV s^–1^ causes a small shift of a few mV at the switching voltage, and hence we have kept the scan rate fixed at 100 mV s^–1^ during this study.^21^ For the PTAA device, the average SET and RESET voltage was 0.24 ± 0.09 V and −0.58 ± 0.06 V, respectively. For the case of the SAM device, the average SET and RESET voltage was 0.10 ± 0.02 V and −0.74 ± 0.10 V, respectively. The transition from the HRS to the LRS is abrupt, indicating filamentary switching. A more detailed discussion regarding the conduction mechanism will be provided later in the manuscript. The magnitude of the high resistance state (HRS) current for both cases is 10^–7^A, while for the low resistance state (LRS) it is 10^–3^–10^–4^ A. Hence the ON/OFF ratio is >10^3^. For the case of PTAA devices, the SET voltage values appear more dispersed than those for SAM-based devices. The switching voltage distribution for both SET and RESET processes is shown in Figure 2c,d for the cases of PTAA and SAM, respectively. The SET voltage distribution is narrower for the case of the SAM devices, and the average SET voltage is reduced compared to that of PTAA devices.

**Figure 2:**
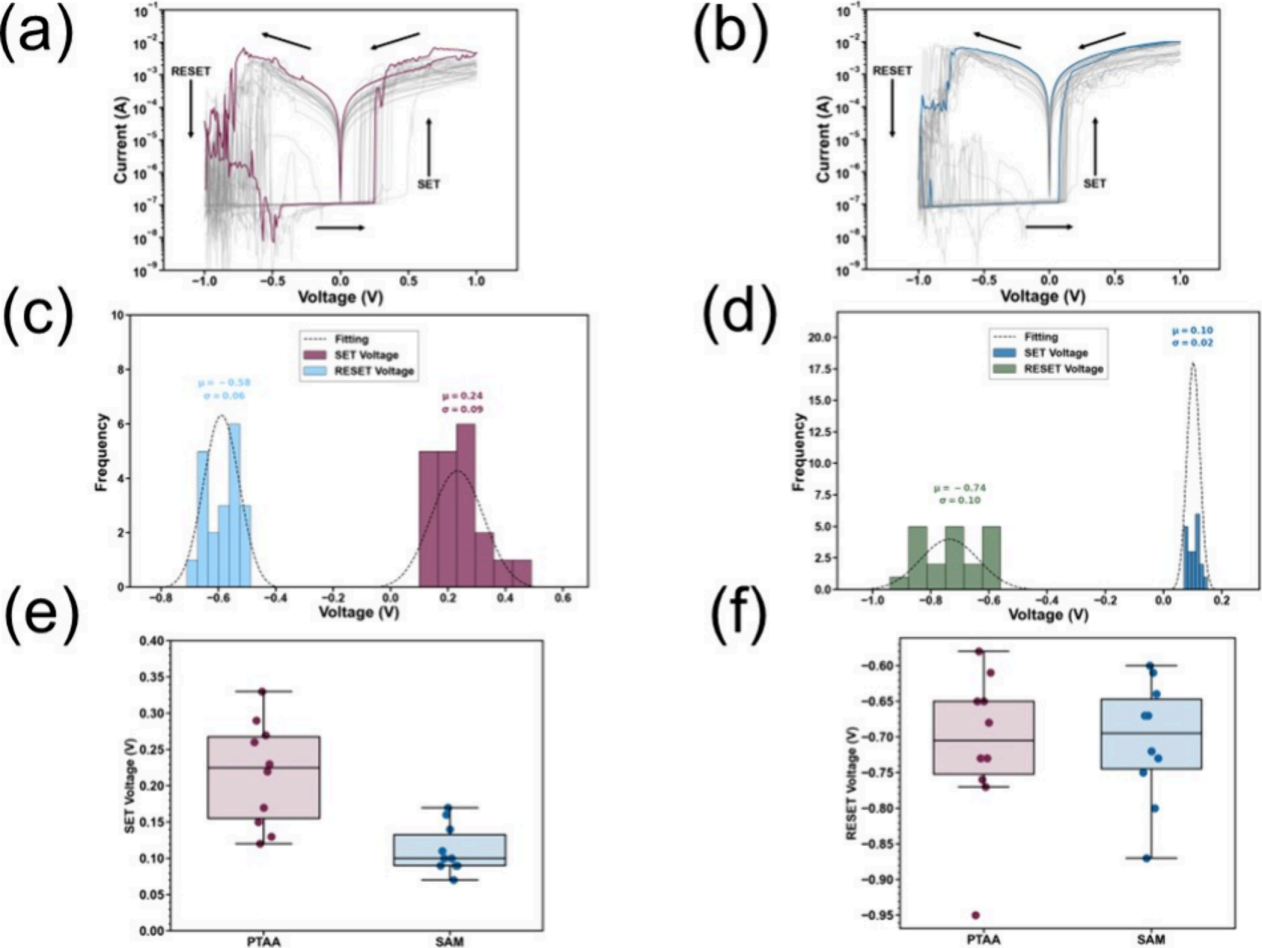
Twenty (20) consecutive current–voltage sweeps of the a) PTAA memory device and b) SAM-based memory device of a single cell. c) Distribution of switching features of the SET and RESET processes for c) PTAA and d) SAM resistive switching memory devices, e) SET and f) RESET switching voltage box plots of 10 cells fabricated with PTAA and SAM as bottom contacts.

In addition to cycle-to-cycle and device-to-device variations, we compared the steady-state switching characteristics in a different batch to examine batch-to-batch variations and confirm the reproducibility of our method. The *I*–*V* characteristics of both PTAA and SAM devices for 20 repetitive cycles from a single cell are illustrated in Figure S3a,c, with their corresponding switching voltage distribution for SET and RESET in Figure S3b,d. Taking into consideration results of both batches (Figure 2 and Figure S3), this leads to the conclusion that there are no significant differences between both HTLs in the RESET voltage; however, we observe a reduction in the SET voltage for the SAM devices. Evidence suggests that SAM molecules can enhance thermal transport across interfaces,^75^ which is beneficial for forming stable conductive filaments. On the other hand, Joule heating can affect the rupture of the conductive filament and hence the RESET process.^76^ SAM molecules have been found to increase thermal transport in a much larger degree.^77^ Excessive thermal transport can cause inconsistencies in the RESET process due to joule heating, which might be the primary reason for the observed variations during multiple, sequential RESET processes in samples from different batches.

To verify further that SAM devices exhibit consistently lower SET voltage values, we measured corresponding *I*–*V* curves in ten different reference and target devices and extracted average values of SET and RESET voltages. The results are illustrated in Figure 2e,f. As in the case of sequential measurements implemented in single devices, the average RESET voltage values of ten different devices appear similar for both HTLs; however, a systematic reduction in SET voltage values is observed for the case of SAM devices. Besides, SAM based devices exhibit a narrower SET value distribution compared to reference devices. A potentially stronger filament formation as a result of the better interface properties of the SAM ultrathin layer with the perovskite film could lead to a lower SET voltage and narrower values distribution. It has been shown that indeed SAM can passivate interfacial defects in PSC.^78−80^

As a next step, we examined the switching characteristics of both devices using measurements with electrical pulses. We proceed by studying the cycling endurance of the MHP memory devices based on PTAA and SAM, by applying thousands of write-read-erase-read cycles in sequence. The endurance waveform consists of the following parameters: write/erase pulses of ±1 V amplitude and 100 ms width, and a read pulse of 20 mV with 2 ms width. The cycling endurance plots with PTAA and SAM MHP devices are included in Figure 3, panels a and b, respectively. Each data point in the endurance plot is the average of all read pulse data points per switching cycle. For PTAA devices, the LRS shows an upward trend and eventually reaches a failure at 8 × 10^2^ switching cycles, while the HRS is constantly at 200 kΩ. Conversely, the SAM device maintains both the LRS and HRS intact after 10^4^ pulsed switching cycles, demonstrating the effect of the SAM bottom contact to improve the device switching durability. Additionally, the device durability was examined in another batch by comparing the cycling endurance of both devices. For PTAA, the amplitude of the SET pulse for cycling endurance measurements was further increased to 1.5 V as an attempt to prevent LRS failure and permanent filament rupture. As indicated in Figure S4a, the cycling endurance was slightly improved at 10^3^ cycles, which is still inferior compared to that of the SAM device. The MHP memory device with the SAM maintains its ON/OFF ratio after 10^4^ switching cycles, as shown in Figure S4b. Box plots with the LRS and HRS values from the endurance of both PTAA and SAM devices are shown in Figure 3, panel c and d, respectively. It is evident that the PTAA LRS has a larger variability compared to that of SAM, and the HRS of the SAM device has a larger variability compared to PTAA. These results corroborate the steady-state IV measurements, and the same trend was observed in another batch (Figure S5).

**Figure 3:**
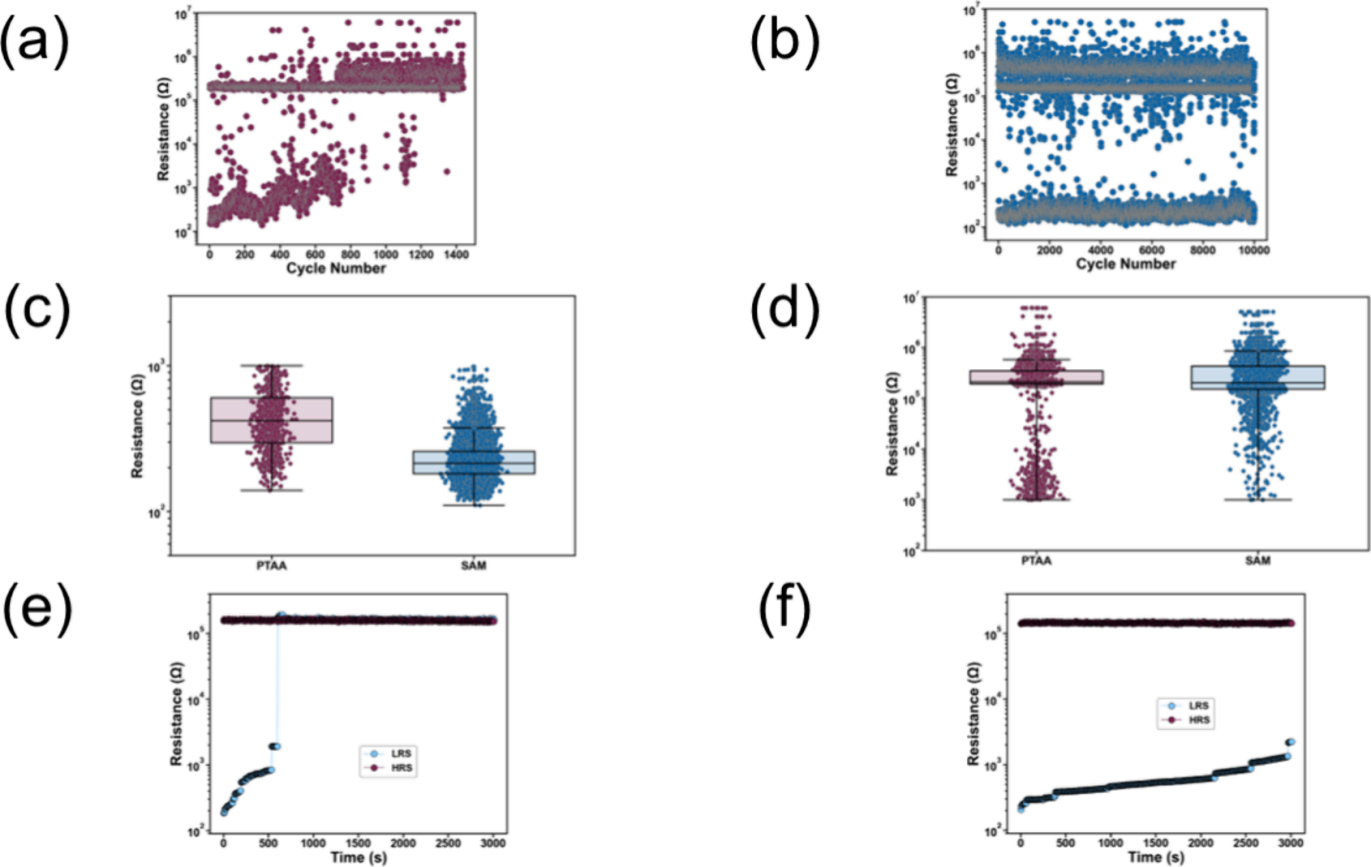
Cycling endurance of typical a) PTAA and b) Meo-2PACz SAM device. Box plots with c) LRS and d) HRS values for the case of PTAA and SAM devices. The state retention of typical e) PTAA and f) SAM RS is also shown.

The nonvolatile characteristics of the PTAA and SAM resistive switching memories were also examined through state retention measurements. The same waveform was used for both devices. The write/erase pulse amplitude was fixed at ±1 V for HRS and LRS, respectively. A read pulse of 10 mV with a duration of 10 ms with a period of 5 s is applied to extract resistance values for both cases. The state retention of PTAA and SAM memory device was monitored for a total period of 3 × 10^3^ s, and the results are depicted in Figure 3, panels e and f, respectively. For the case of PTAA, the device failure occurs at 5 × 10^2^ s with an abrupt transition of the LRS to the HRS, which leads to permanent filament rupture and is the predominant failure mechanism in our system.^81,82^ The HRS has remained intact close to 200 kΩ. For the SAM device, the state retention is maintained during the 3 × 10^3^ s time interval. The LRS increases from 470 Ω initially to 2.8 kΩ at the end of the measurement. Like the PTAA devices, the HRS was maintained close to 200 kΩ. These results are in agreement with the endurance behavior of the two HTLS, where the device failure and filament rupture occur much faster for the case of PTAA and can be attributed to the worse HTL/perovskite interface compared to the SAM devices. The results agree with the steady-state current–voltage characteristics, where the SET process is more stable and occurs at a reduced electric field for the SAM device. These findings highlight the potential of the SAM bottom contact to improve the nonvolatile behavior of the MHP resistive switching memory. We can therefore conclude that the replacement of PTAA with SAM molecules improved both cycling endurance and retention of the nonvolatile MHP memory device.

We proceed by further analyzing the *I*–*V* characteristics of the PTAA and SAM MHP memristive devices to identify the conduction mechanism responsible for the memristive switching. In MHP memory devices, Schottky barrier modification^83,72^ and filamentary switching are the predominant mechanisms that govern resistive switching.^84^ Two main causes are responsible for filament formation/rupture that occurs in MHP memories: the first is through metal cation diffusion between the top and bottom electrodes induced by an active metal (such as Ag) by the electrochemical metallization mechanism. In MHP devices the low activation energy for halide migration^85−87^ can also lead to conductive filaments formed by halide vacancies through the valence change mechanism, which is the second cause for filament formation. The choice of top electrode can severely impact the mechanism responsible for resistive switching.^88^ In our system, we report the coexistence of both metallic and halide vacancy filaments, which has also been illustrated previously in MHP memristive devices.^89^ The abrupt transition from LRS to HRS is an indication of the formation of metallic filaments. In addition, we have identified in our previous reports a light-induced ion-vacancy recombination effect which increases the LRS, thus confirming the presence of filaments induced by ion vacancies.^90−92^ A simplified illustration of the conduction mechanism of our MHP device is illustrated in Figure 4a for the ON state and in Figure 4d for the OFF state. Initially, the halide ions are randomly distributed through the perovskite layer. After filament formation, the positive voltage applied on the Ag top electrode attracts I^–^, leaving behind halide vacancies (*V*_I_,_Br_), which eventually form a conductive path between the Ag top electrode and the ITO bottom electrode. In addition, metallic Ag cations diffuse toward the ITO bottom electrode. When a negative bias is applied on the Ag top electrode, the ions drift away from the top electrode, filling back the vacancies. This leads to filament rupture, as in the case of metallic filaments. Comprehensive analysis of the complex filamentary switching mechanism that involves interactions of metallic and vacancy filaments can be found in these studies.^93−95^ To quantitatively demonstrate the filamentary switching mechanism, we examined the *I*–*V* characteristics of the PTAA and SAM devices in the LRS plotted in a log–log scale, as shown in Figure 4b,c. For both cases, the current varies linearly with voltage. Linear fitting shows linear behavior with a slope of 1.07 and 1.04 for PTAA and SAM devices, respectively. The resulting ohmic conduction in the LRS confirms the presence of filamentary switching for MHP resistive switching devices. The same analysis was performed in HRS by plotting ln(*I*) as a function of V^1/2^, as presented in Figure 4e,f for the case of PTAA and SAM, respectively. The relationship between ln(*I*) and *V*^1/2^ is linear, and therefore we conclude that Shottky emission is the predominant conduction governing the HRS.^83,94,96^

**Figure 4:**
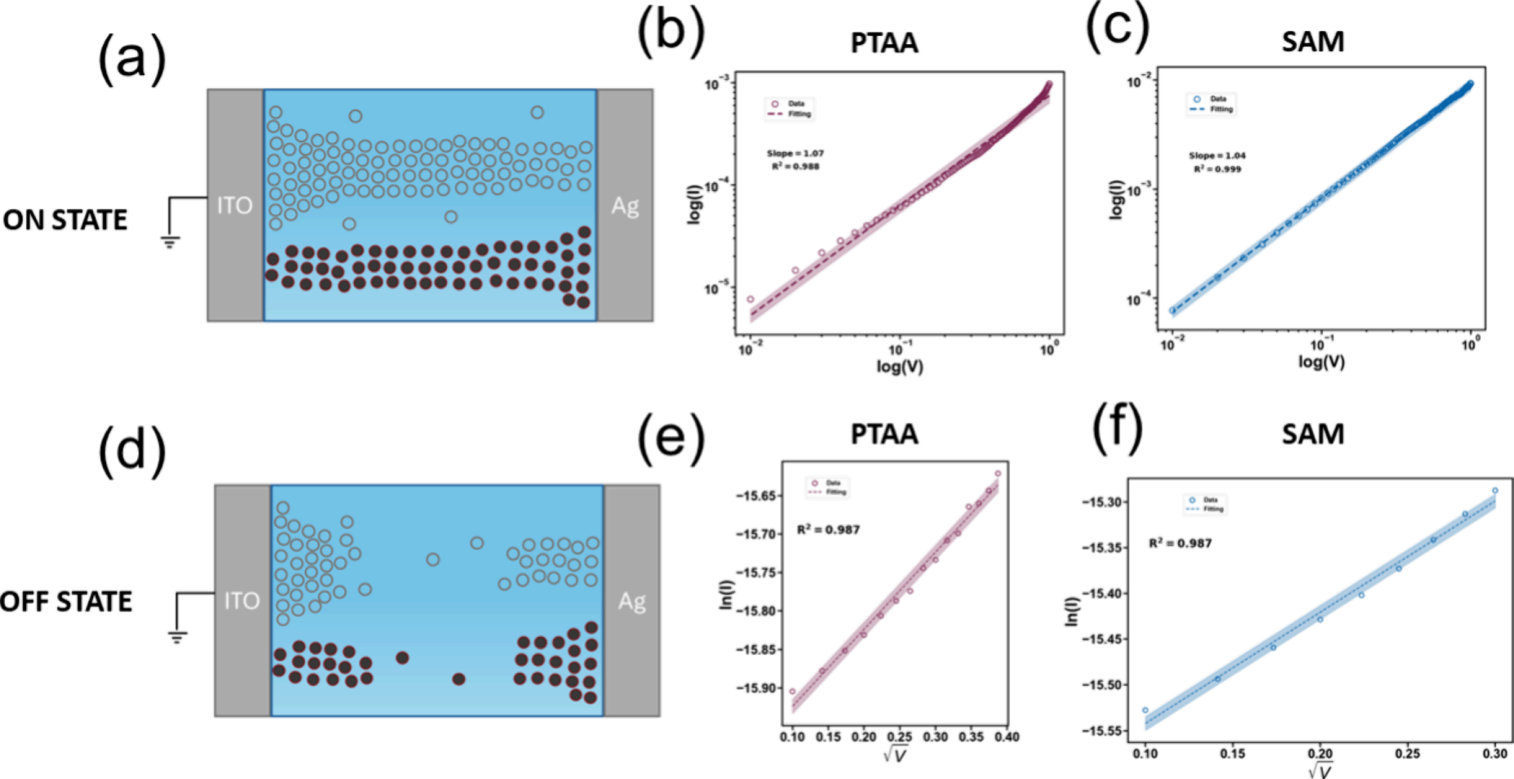
Schematic illustration of filamentary switching during the a) LRS (ON state) and d) HRS (OFF state). Color-filled circles represent metal cations, while empty circles represent halide ion vacancies. Log–log plots for the b) PTAA and c) SAM device in the LRS. and ln *I*-V^1/2^ plots in the HRS for e) PTAA and f) SAM RS device.

In summary, we have demonstrated a cost-effective approach to improve the performance of MHP perovskite resistive switching memories by replacing the expensive PTAA HTL with MeO-2 PaCZ SAM. The study is based on a photovoltaic memristive device with an ON/OFF ratio of >10^3^ which operates at low electric fields. The comparison between PTAA and SAM devices is based on either multiple sequential memristive current–voltage characteristics of single devices or average data of multiple reference and targeted devices. In both cases, resistive switching memory devices based on SAM exhibit improved performance, having reduced average SET voltage values and narrower statistical variation compared to reference devices with PTAA. The reduction of the SET voltage for the case of SAM devices is a result of a stronger filament formation due to the improved SAM/perovskite interface that also led to the suppressed variability of SET voltage values. The improved performance of targeted samples is also indicated by pulsed measurements, such as retention and cycling endurance. The conduction mechanism for both HTL materials was identified as the formation/rupture of conductive filaments induced by metal cations and halide vacancies, a process during which excessive heat is generated, affecting SET/RESET processes. Overall, the proposed manufacturing approach is compatible with upscalable device fabrication enabled by industrially compatible printing methods on flexible substrates with the potential for high throughput production.

## Supporting Information Available

Materials, device fabrication and characterization, AFM images of the perovskite, PTAA and SAM thin films, 20 current–voltage curves for PTAA and SAM devices from a different batch with their switching voltage distributions, cycling endurance of PTAA and SAM devices, box plots with resistance values of the endurance from another batch (PDF)
