# Supplementary material for: Sustainable Mixed-Halide Perovskite Resistive Switching Memories Using Self-Assembled Monolayers as the Bottom Contact
Source: J Phys Chem Lett. 2024 Jul 22;15(30):7635–44. doi: 10.1021/acs.jpclett.4c01664 (PMC11299189; doi:10.1021/acs.jpclett.4c01664)
Supplement: Supplementary file 1 — jz4c01664_si_001.pdf [file jz4c01664_si_001.pdf]

Supporting Information

Sustainable mixed-halide perovskite resistive switching memories using self-assembled monolayers as the bottom contact

Michalis Loizos[1], Konstantinos Rogdakis[1,2], and Emmanuel Kymakis[1,2*]*

[1] Department of Electrical & Computer Engineering, Hellenic Mediterranean University (HMU), Heraklion 71410, Crete, Greece

[2] Institute of Emerging Technologies (i-EMERGE) of HMU Research Center, Heraklion 71410, Crete, Greece

Experimental Section

Materials

Chlorobenzene (CB, anhydrous, 98%), N,N-dimethylformamide (DMF, anhydrous, 99.8%), dimethyl sulfoxide (DMSO, anhydrous, ≥99.9%), toluene (anhydrous, 99.8%), Ethanol (EtOh, anhydrous), cesium iodide (CsI, 99.999%, trace metals basis), rubidium iodide (RbI, 99.9% ,trace metals basis), 2,9-dimethyl-4,7-diphenyl-1,10-phenanthroline (bathocuproine,BCP,96%), were purchased from Sigma Aldrich. 2-propanol (IPA, Extra Dry, 99.5%) was purchased from Acros Organics. Lead iodide (PbI_2, trace metals basis, 99.99%), lead bromide ($PbBr_2$ >98.0%), and [2-(3,6-Dimethoxy-9H-carbazol-9-yl)ethyl]phosphonic Acid (MeO-2PACz, >98%) were purchased from TCI. Formamidinium Iodide (FAI) and methylammonium bromide (MABr) were purchased from Dyenamo. Phenyl-C61-butyric acid methyl ester ($PC_{61}BM$, 99%) was purchased from Solenne BV. Poly[bis(4-phenyl)(2,4,6-trimethylphenyl)amine] (PTAA, Mw = 20–70 kDa) was purchased from Solaris Chem.

Device Fabrication and characterization

Glass/ITO substrates (Naranjo) are cleaned by ultrasonication bath in water, acetone, and isopropyl alcohol , each step for 10 min. The substrates were then dried in N_2 flow, following an oxygen plasma treatment for 5 min, and were then transferred to a N_2-filled glovebox for further processing. For PTAA HTL, 2 mg were dissolved in 1ml toluene, and was spin-coated on the glass/ITO substrates at 6000 rpm for 30 following annealing at 100 °C for 10 min. For MeO-2PACz, 0.5mg were dissolved in 1ml ethanol. The solution is then spin-coated at 3000 rpm for 30s, following annealing at 100°C for 10min. The perovskite precursor solution was prepared according to previous reports. The final composition of the four cation RbCsFAMA solution is $Rb_{0.04}Cs_{0.05}(FA_{0.85}MA_{0.15})_{0.91}Pb(I_{0.85}Br_{0.15})_3$. The solution is dynamically spin-coated at 6000 rpm for 45 s. Afterward, 200 µl of

chlorobenzene was poured on the centre of the rotating substrate 20 s before the end of the spinning process, following annealing at 100 °C for 45 min. Then, 20 mg ml^{-1} PCBM in chlorobenzene are spin-coated at 2000rpm for 60s. Next, 0.5 mg ml^{-1} of BCP in IPA are dynamically spin-coated on the substrates. Finally, 100 nm Ag were thermally evaporated under a high vacuum (10^{-6} mbar). The active area of the memristive device is 4 mm^2. The steady-state characteristics of the resistive switching memories were measured using the ARKEO commercial equipment provided by Cicci Research. The I-V characteristics of the resistive switching memories were measured by applying bias on the Ag top electrode, while the ITO bottom electrode was grounded. The voltage was swept from 1.2 or 1 V to -1V, with 100 mV s^{-1} scan rate and 10mV step. A compliance current of 10mA was used to protect the devices. Pulsed characterization was performed through a customized interface developed by Cicci Research, which allows the custom design of electric pulses. All measurements were performed in ambient atmosphere at controlled humidity (30-40%) at 25°C. AFM topography of the PTAA, SAM, and perovskite film with 20x20μm,20x20μm and 5x5μm size respectively was recorded by the XE7 microscope from Park Systems. The topography profiles were analyzed for thickness measurement. Thickness measurement for both HTLs was estimated by analyzing the height profile of 5 lines across the topography images and obtaining the average of these values as the thickness estimation. Analysis was performed using the Gwyddion free software.

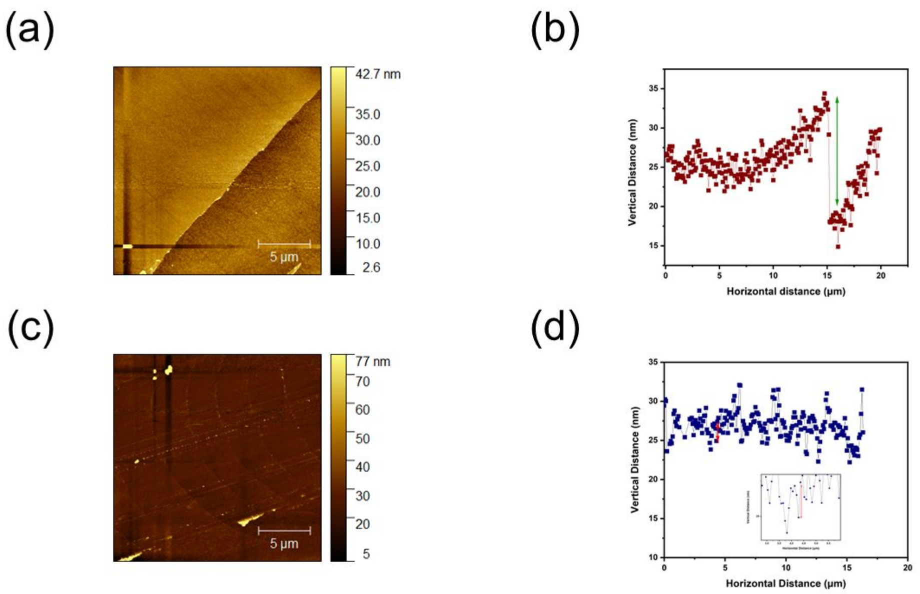

Figure S1 20x20µm AFM image of a) PTAA ,b) SAM thin film with the height profiles b) and d) respectively that were used to obtain the thickness.

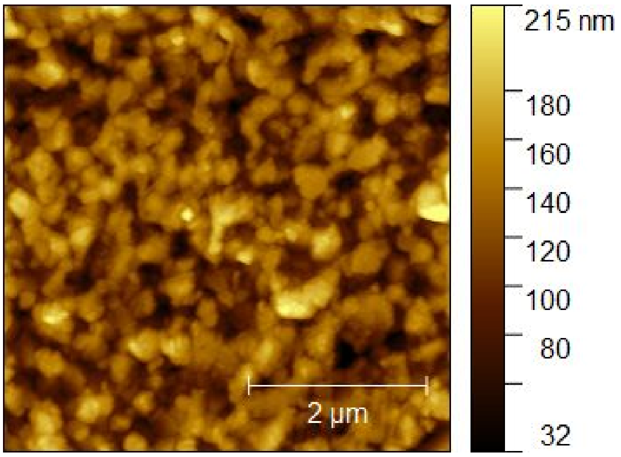

Figure S2 5x5µm AFM image of the perovskite layer

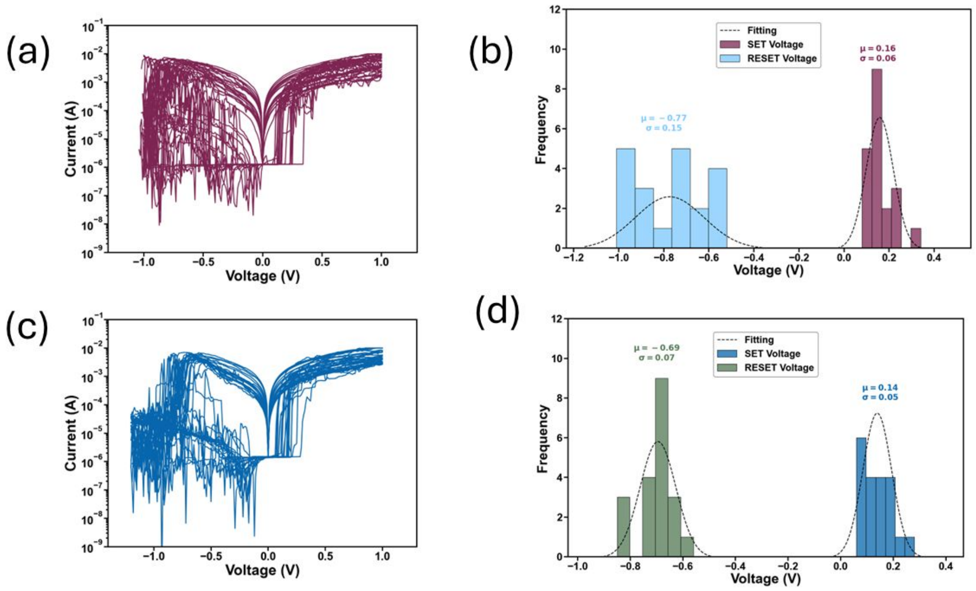

Figure S3 a) 20 I-V curves of PTAA-based devices from another batch with b) switching voltage distribution, c) 20 I-V curves of perovskite resistive switching memory with SAM as the HTL from another batch and its d) switching voltage distribution

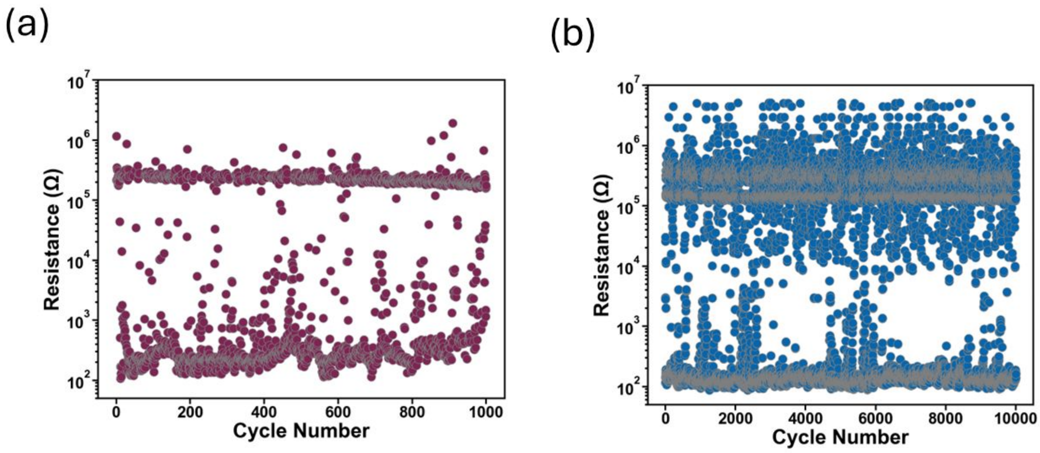

Figure S4 Endurance plots from another batch for a) PTAA (Set pulse width =1.5V) and b) Meo-2PACz device (SET pulse width=1V).

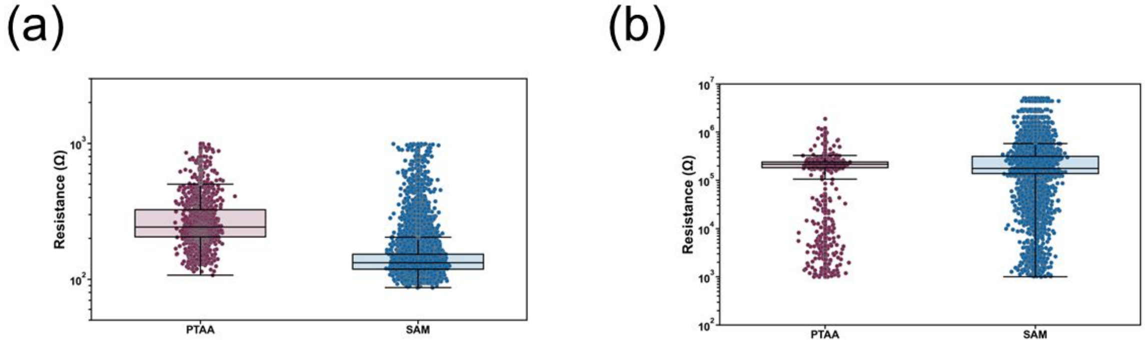

Figure S5 Box plots with the obtained a) LRS and b) HRS values from the batch of figure S4 for both PTAA and SAM.
